# Supplementary material for: Inclusion of persons living with disabilities in a district-wide sanitation programme: A cross-sectional study in rural Malawi
Source: PLOS Glob Public Health. 2024 Aug 29;4(8):e0003005. doi: 10.1371/journal.pgph.0003005 (PMC11361574; doi:10.1371/journal.pgph.0003005)
Supplement: S1 Text — (DOCX) [file pgph.0003005.s001.docx]

**Table A. Full regression results for the association between having a household member with a disability and being invited to triggering.**


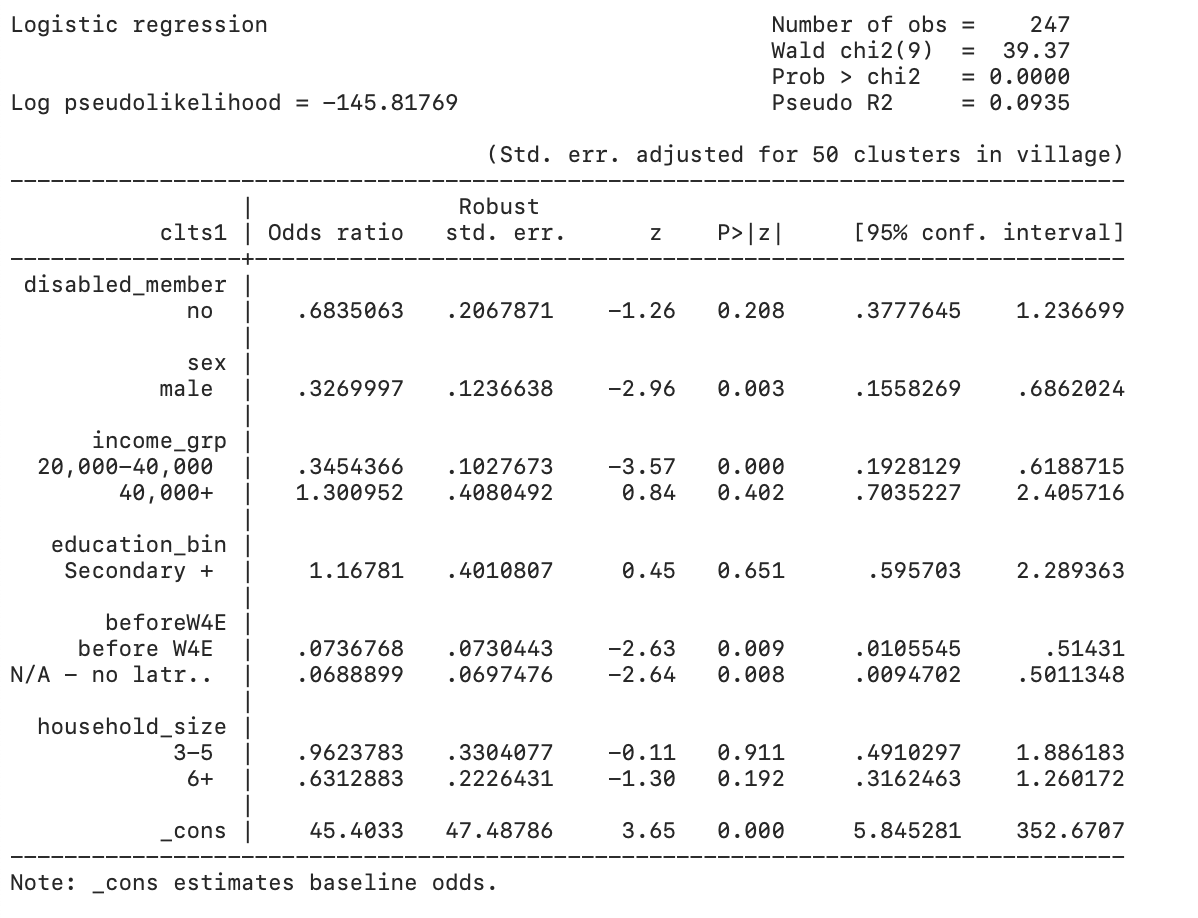


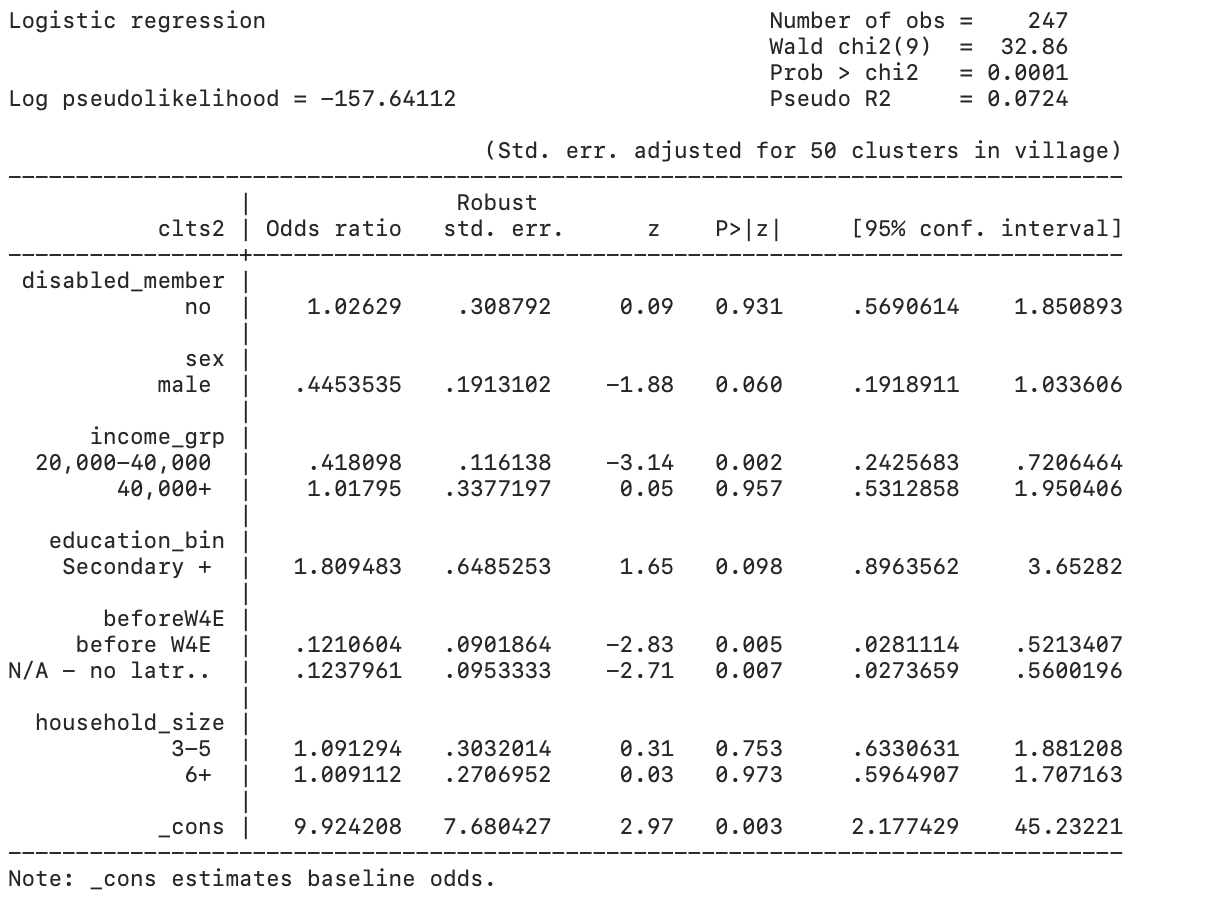
**Table B. Full regression results for the association between having a household member with a disability and attending triggering.**


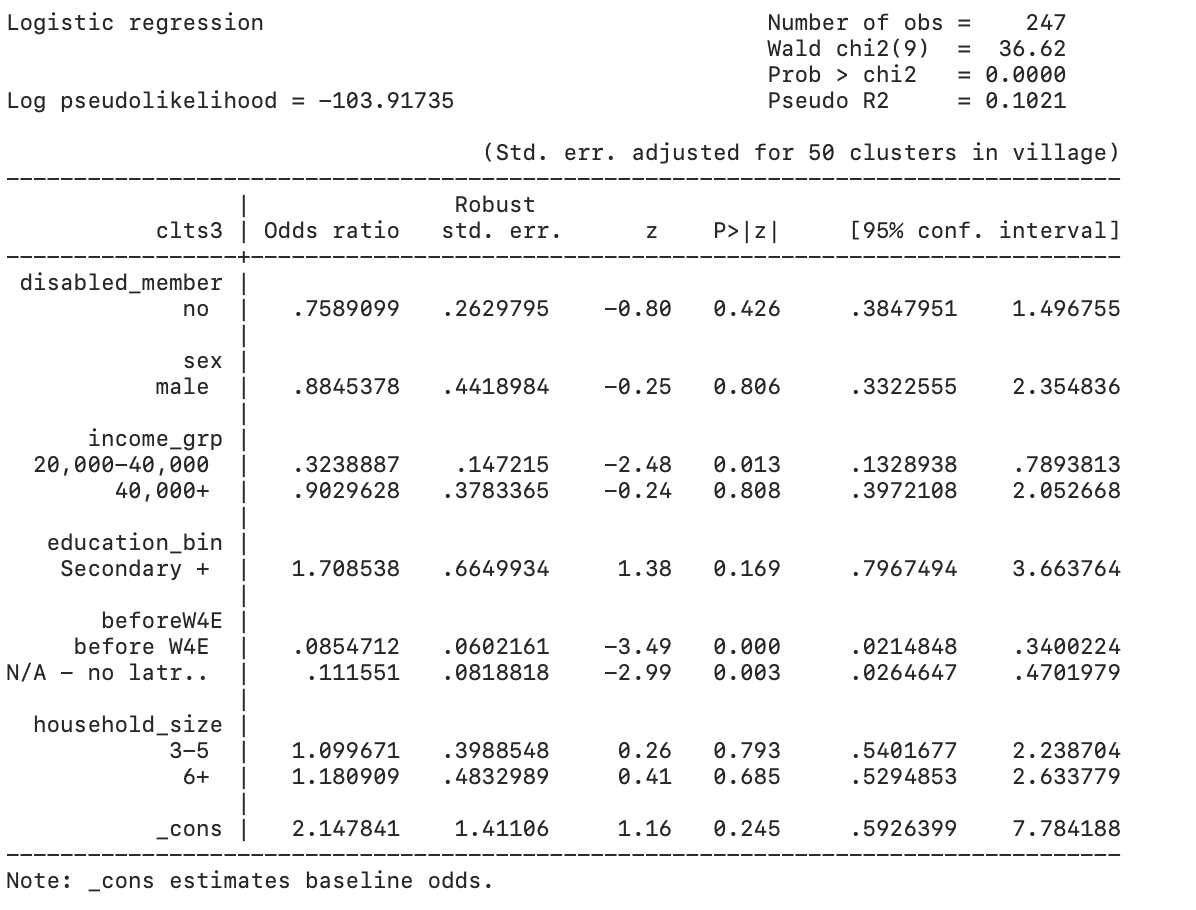
**Table C. Full regression results for the association between having a household member with a disability and participating in the transect walk.**


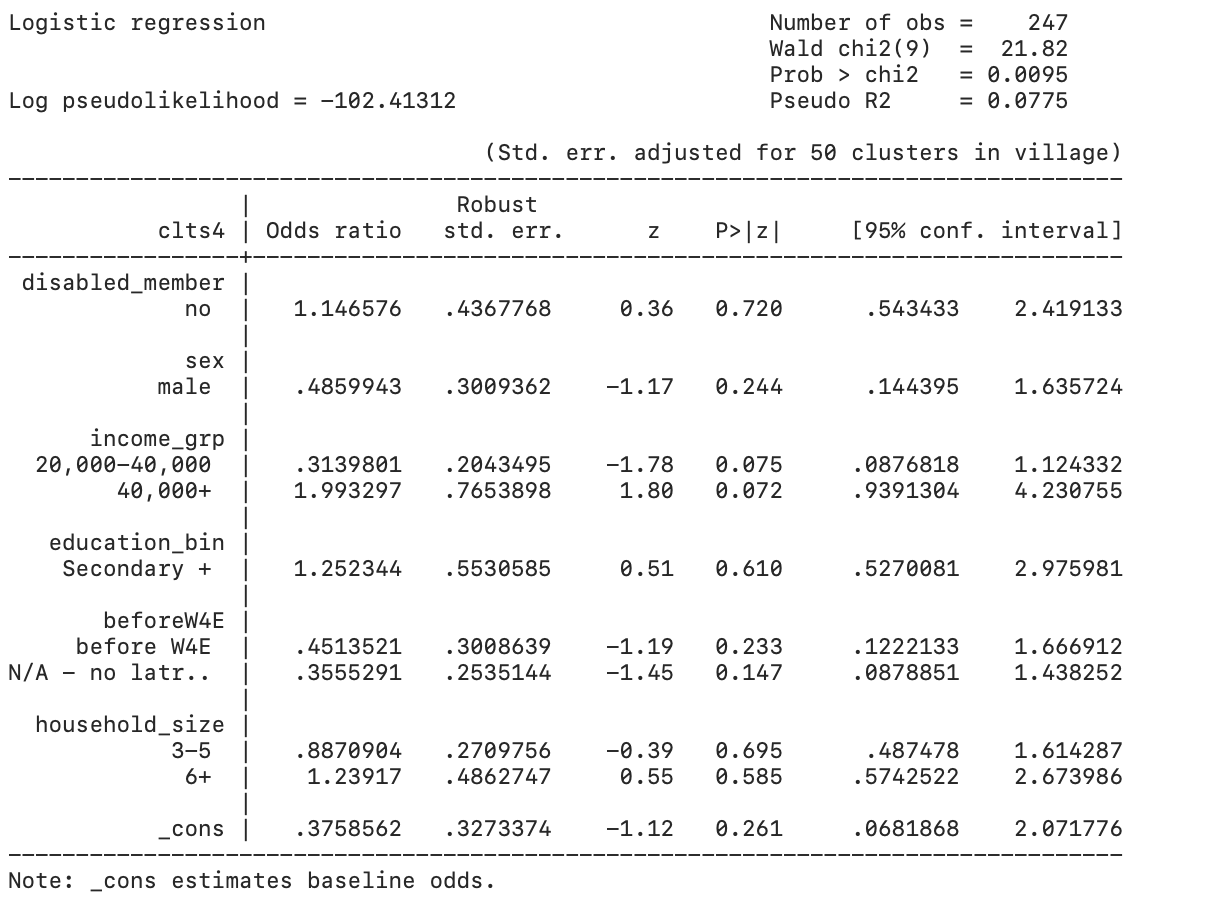
**Table D. Full regression results for the association between having a household member with a disability and participating in community mapping.**


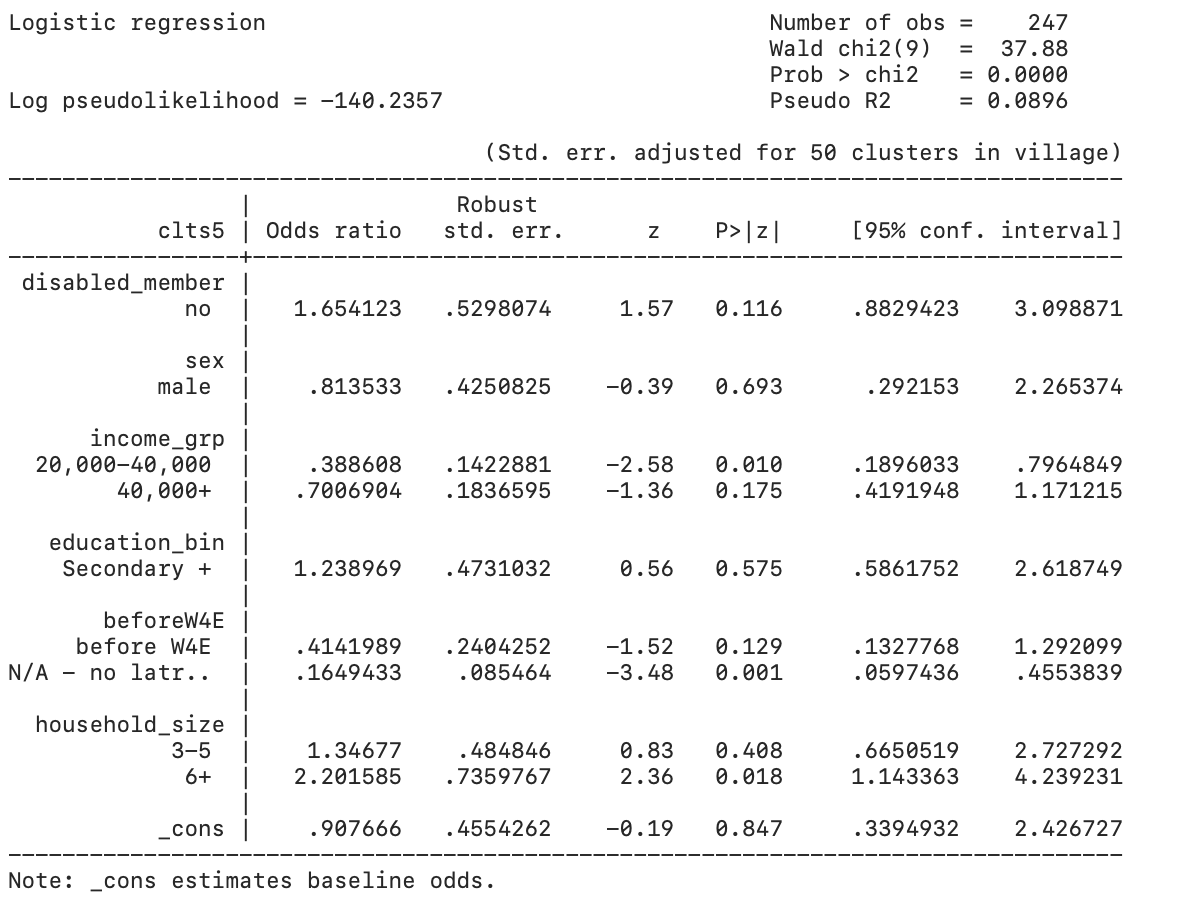
**Table E. Full regression results for the association between having a household member with a disability and feeling they could give input.**


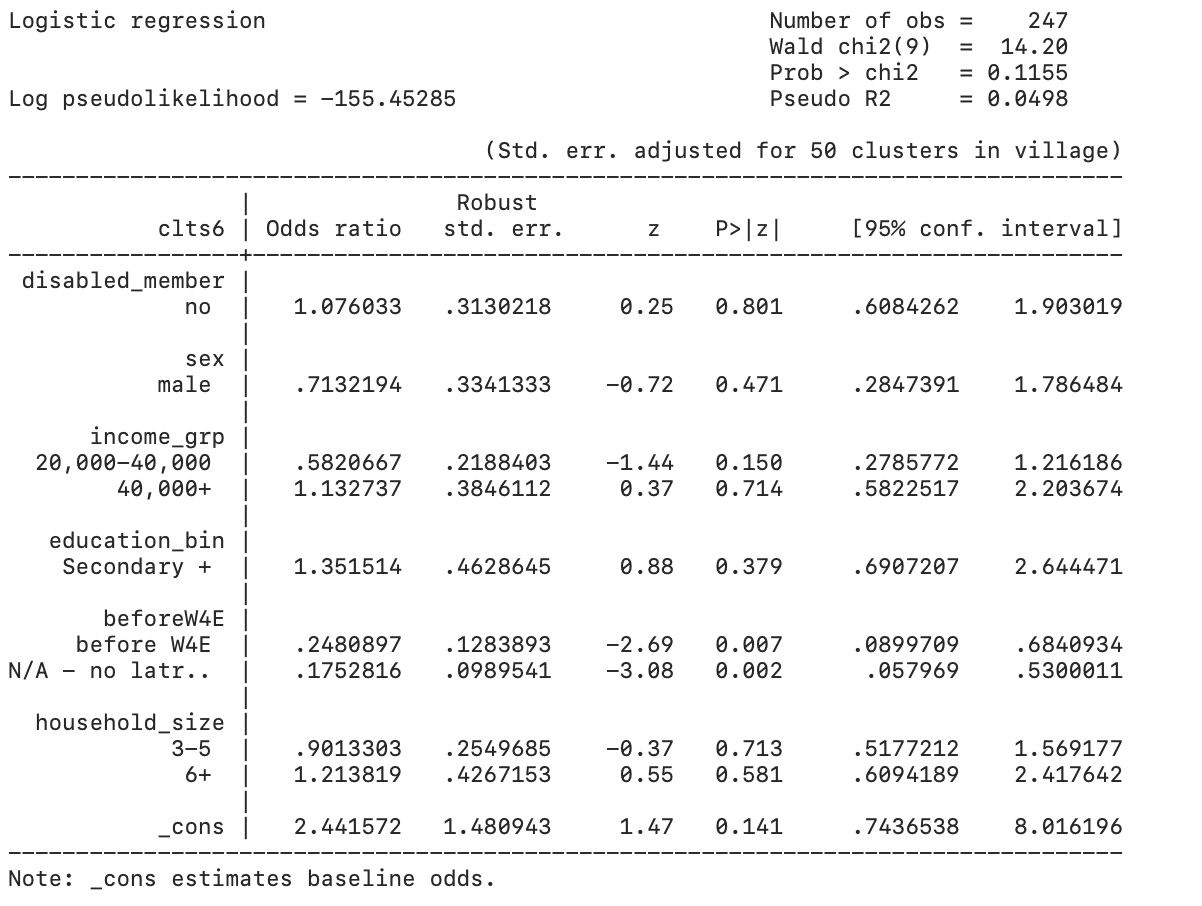
**Table F. Full regression results for the association between having a household member with a disability and participating in community action planning.**


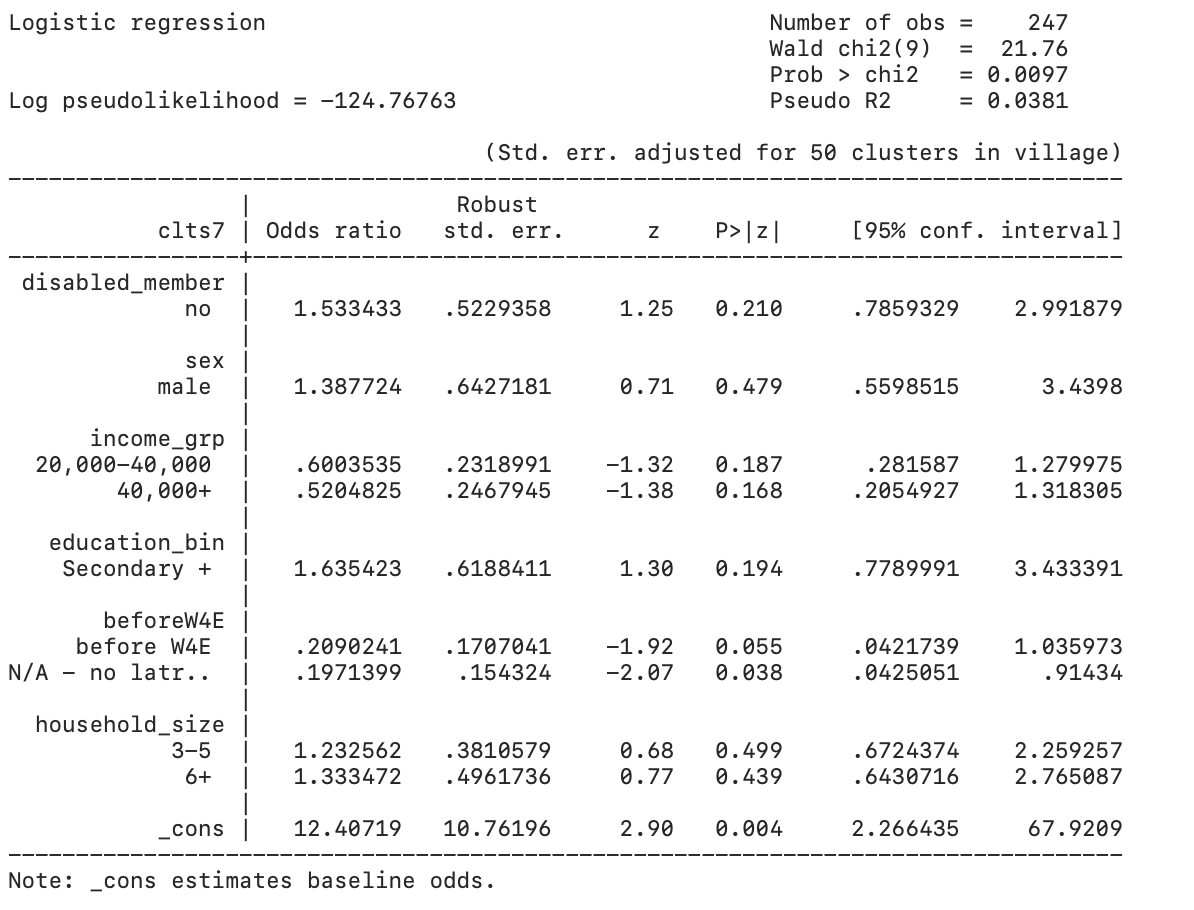
**Table G. Full regression results for the association between having a household member with a disability and being visited to discuss latrine construction/use.**
