# Supplementary material for: Inclusion of persons living with disabilities in a district-wide sanitation programme: A cross-sectional study in rural Malawi
Source: PLOS Glob Public Health. 2024 Aug 29;4(8):e0003005. doi: 10.1371/journal.pgph.0003005 (PMC11361574; doi:10.1371/journal.pgph.0003005)
Supplement: Attachment — Submitted filename: Review.pdf [file pgph.0003005.s005.pdf]

Davies et al. 2024

The authors present a clear, thorough description of a strong study on an important topic. The nature of the topic presents challenges for obtaining samples large enough for statistical precision. Despite this, the authors uncovered statistically detectable disparities in CLTS participation for individuals living with disabilities. My specific comments point out areas for potential improvement in clarity of the analysis and manuscript. After some minor revisions, this manuscript can be a strong contribution to the literature.

- **Introduction**
    - Lines 68-69:
        - I take "…viewing disablement as a consequence of external factors in addition to personal impairments" to indicate that personal impairments do not necessarily cause disability unless external factors limit one's ability to participate fully and equally. However, I think the sentence could also be read as suggesting external factors can cause a physical disability (e.g., resulting from an accident). If my interpretation is correct, this is an important point and motivates your study well, so I suggest expanding your point for clarity.
    - Lines 121-122:
        - "People living with disabilities are often under-estimated by their communities that think they are incapable of participating in community meetings."
        - I initially thought that "People living with disabilities are often under-estimated" meant that the *number* of people living with disabilities is under-estimated, so I couldn't figure out how the rest of the sentence fit. This may be specific to me, but I suggest rephrasing to clarify what that communities are under-estimating people living with disabilities' ability to participate in community meetings.
    - Overall:
        - The introduction was clear and clearly explained the motivations for and importance of the study.
        - More details on how sanitation infrastructure can hinder or expand access to people living with disabilities could make the manuscript stronger. As someone who doesn't know a lot about the specific infrastructure available, I feel that I still don't have a great grasp on what options exist and how those vary by income/cost.
        - You mention "low-cost adaptations to improve the accessibility of latrines", and I think describing those briefly would be helpful.

- **Methods**
  - Lines 180-182:
    - "Despite efforts to engage with local organizations of persons with disabilities (OPD) for identification and recruitment, we were unable to secure participation."
    - I appreciate your intention of working with organizations that serve this specific population, and I appreciate you including this sentence to show your intention despite it not working out. I am, however, concerned that it leaves too much to the imagination. My first reaction was wondering if local organizations didn't approve of your work or approach for some reason. Could you provide more detail about what happened? I am not sure what would be appropriate for the scientific manuscript, so I am more interested in hearing your thoughts than prescribing a specific recommendation.
  - Lines 172-178, 210-212, 243-245:
    - It wasn't clear to me as I read the methods how households without a person with a disability were included in the study.
    - At lines 172-178, it seems the 200 households are part of your analysis, but then at lines 210-212 the survey is described only for households with a member with a disability. At lines 243-245, I did not understand who the reference group for regression would be.
    - I think this can be clarified easily. I suggest specifying under 'Sampling Procedure' that the 200 households from the broader study are the reference group for your study, and under 'Data Collection' clarifying what surveys were completed by the 200 control households.
    - Note that during my review Figure 2 was located at the end of the document, so I did not see it as I read the Methods. Although Figure 2 makes the sampling clear, I think it would be beneficial to include that in the text as well.
  - Lines 247-248:
    - There are multiple ways to account for clustering, e.g., cluster-robust standard errors, random intercepts, etc. Please specify your approach briefly.
  - Line 253:
    - Presumably individual-level information is still clustered at the village level, as well as household. If you think this is correct, clustering should be accounted for at the highest level. In this case, that would be the village. Fortunately, accounting for clustering at the highest level takes care of clustering at lower levels. So only one correction is needed.
    - The following reference explains that this is actually not always the case. I expect the village-level to still be ideal in your case, but please explain if not.

        - Bottomley, C., Kirby, M.J., Lindsay, S.W. et al. Can the buck always be passed to the highest level of clustering?. BMC Med Res Methodol 16, 29 (2016). https://doi.org/10.1186/s12874-016-0127-1
    - Lines 256-257:
        - Can you explain why univariate regression was used for this subset of models, but not models of program participation?
        - I would think that age, sex, and income would apply to all models equally.
- **Results**
    - Lines 283-288:
        - I think it would be informative to also describe the demographics of households from the random sample (~200 households), which represents the broader study population, and the purposive sample (~50 households). One-hundred percent of households in the purposive sample includes someone with a disability, for example, so I think it is more noteworthy to report that ~30/200 households from the random sample included an individual with a disability. The results already provided can still be included to describe the total sample, but I recommend including subgroup results as well.
            - Note that I believe the structure of Table 1 and Table 2 (separating any household/individual with a disability vs. without) is fine and I do not recommend separating the detailed results by sample.
    - Table 3:
        - Can you please that I have the correct interpretation of the aOR for invitation to the triggering event? Households with no member with a disability were 32% less likely (aOR = 0.68, 95% CI 0.38, 1.24) to be invited to the triggering event compared to households with a member with a disability.
            - I want to clarify because I am surprised at the direction of the aOR, considering the proportion invited is larger for households with no member with a disability. I don't doubt it is plausible after adjusting for covariates, but it surprises me so I want to be sure.
    - Overall:
        - Did you consider gender as a potential effect modifier rather than a confounder?
            - It seems plausible to me that having a disability affects community participation, social connection, etc. differently between men and women.
            - Also, since individuals without disabilities are so much more likely to be female (92%) than individuals with disabilities

(44%), adjusting for gender may not be enough to really account for the gender distributions. I have seen situations where an adjusted estimate masks differences for heavily skewed covariates.

- I would be interested in seeing how CLTS participation differs between men with disabilities and women with disabilities. The sample size may not be large enough to separately fit regression models by gender, but if sample size weren't an issue I would be interested in that as well.

- **Discussion**
    - Overall:
        - It is unclear to me if CLTS activities were *supposed to* cover issues specific to individuals with disabilities, such as low-cost technologies.
        - Since this study was conducted in partnership with World Vision, I think it would be helpful to discuss whether disability-relevant issues were considered before implementation. The results should be interpreted differently depending on training. If CLTS facilitators failed to cover information that they were trained to include, there is a significant issue with implementation. If CLTS facilitators were not specifically instructed to cover disability-relevant information, the results demonstrate the need to specifically incorporate them into training.
