# Supplementary material for: Inclusion of persons living with disabilities in a district-wide sanitation programme: A cross-sectional study in rural Malawi
Source: PLOS Glob Public Health. 2024 Aug 29;4(8):e0003005. doi: 10.1371/journal.pgph.0003005 (PMC11361574; doi:10.1371/journal.pgph.0003005)
Supplement: Attachment — Submitted filename: Response to Reviewers.pdf [file pgph.0003005.s006.pdf]

20 June 2024

Dear Editor,

Thank you for inviting us to submit a revised draft of our manuscript, "**Inclusion of persons living with disabilities in a district-wide sanitation programme: a cross-sectional study in rural Malawi",** to PLoS Global Public Health. We thank you and the reviewers for providing insightful feedback on how to strengthen our paper. We have amended the manuscript to address these comments, with detailed responses to each comment provided on the following page.

Thank you for considering our manuscript for publication. We look forward to your response.

Sincerely,

Katherine Davies

Reviewer comment	Response	Action/revision
**Editor/Journal Requirements**		
Please include a complete copy of PLOS' questionnaire on inclusivity in global research in your revised manuscript.	Thank you, this has been uploaded as supporting information with a sentence refencing it in the methods section.	**Added sentence:** "Additional information regarding the ethical, cultural, and scientific considerations specific to inclusivity in global research is included in the supporting information." (page 12, lines 275-277)
Please review the reference list to ensure that it is complete or correct. If you have cited papers that are retracted, please include the rationale for doing so in the manuscript text or remove these references and replace them with relevant current references. Mention any changes to the reference list in the rebuttal letter. If you need to cite a retracted article, indicate the article's retracted status in the reference list and include a citation and full reference for the retraction notice.	The reference list has been reviewed to ensure it is complete and correct. No papers that are retracted have been cited. Two additional references have been added: [51] and [52].	Two references have been added [51] and [52].
**Reviewer 1**		
**Minor Edits**		
Page 12 line 213 states Washing Group Short Set (WG-SS) which I think should be Washington Group Short Set (WG-SS).	Thank you, this has been amended to read "Washington Group Short Set". This sentence was restructured as per reviewer 2's comments below.	**Amended sentence:** "To determine if anyone in the household had a disability, the Washington Group Short Set (WG-SS) of questions on functioning were then administered with the primary household respondent [45]." (page 9, lines 220-221)
For reading clarity, I suggest format tables 1 and 2 to have the percentages (%) closer to relevant number within each column, as currently formatted they are closer to the numbers for the subsequent column.	Tables 1 and 2 have been reformatted to have the percentages in brackets closer to the relevant number in each column. Rows with totals have also been deleted make the tables less cluttered. Decimal places have been removed from percentages.	Tables 1 and 2 have been updated (pages 14-18, lines 318-322)

**Reviewer 2**		
**Introduction**		
Lines 68-69: I take "…viewing disablement as a consequence of external factors in addition to personal impairments" to indicate that personal impairments do not necessarily cause disability unless external factors limit one's ability to participate fully and equally. However, I think this sentence could also be read as suggesting external factors can cause a physical disability (e.g., resulting from an accident). If my interpretation is correct, this is an important point and motivates your study well, so I suggest expanding your point for clarity.	Thank you, this sentence has been amended to clarify the meaning of "external factors".	**Amended sentence:** "The World Health Organisation's (WHO) International Classification of Functioning, Disability and Health (ICF) framework provides a bio-psycho-social model of disability, viewing disablement as a consequence of external factors that limit one's ability to participate fully in society, in addition to personal impairments [3]." (page 3, line 66)
Lines 121-122: "People living with disabilities are often under-estimated by their communities that think they are incapable of participating in community meetings." I initially thought that "People living with disabilities are often underestimated" meant that the number of people living with disabilities are often underestimated, so I couldn't figure out how the rest of the sentence fit. This may be specific to me, but I suggest rephrasing to clarify that communities are under-estimating people living with disabilities' ability to participate in community meetings.	Thank you, this sentence has been re-phrased to make it clear that communities under-estimate their ability to participate in community meetings rather than under-estimate the number of people living with disabilities.	**Amended sentence:** "Communities often under-estimate the ability of people living with disabilities to participate in community meetings." (page 5, lines 120-121)
More details on how sanitation infrastructure can hinder or expand access to people living with disabilities could make the manuscript stronger. As someone who doesn't know a lot about the specific infrastructure available, I feel that I still don't have a great grasp on what options existing and how those vary by income/cost. You mention "low-cost adaptations to improve accessibility of latrines", and I think describing those briefly would be helpful.	Thank you, a brief description of the barriers to sanitation access and low-cost adaptations have been added to the introduction.	**Amended:** "Barriers to sanitation access can include infrastructural factors (uneven terrain, high steps or narrow entrances), discrimination and exclusion from participating in community sanitation meetings." (page 3, line 70)<br><br>**Amended:** The inclusion of people living with disabilities in community programmes involves action by facilitators to ensure they are able to participate, to raise community awareness of the barriers to accessing sanitation, and to disseminate information about low-cost adaptations to improve the accessibility of latrines such as ramps, wide entrances, handrails, and moveable seats [36, 38, 39]." (page 6, lines 126-127)

**Methods**		
Lines 180-182: “Despite efforts to engage with local organisations of persons with disabilities (OPD) for identification and recruitment, we were unable to secure participation.”. I appreciate your intention of working with organisations that service this specific population, and I appreciate you including this sentence to show your intention despite it not working out. I am, however, concerned that it leaves too much to the imagination. My first reaction was wondering if local organizations didn’t approve of your work or approach for some reason. Could you provide more detail about what happened? I am not sure what would be appropriate for the scientific manuscript, so I am more interested in hearing your thoughts than prescribing a specific recommendation.	Thank you for raising this. I have discussed this with the local team and clarified why we did not secure participation.<br><br>The local OPD operated at a regional level so it was felt the district council social welfare office would be best placed to aid us in the study. This has been clarified in the manuscript.	**Amended:** “Organisations of persons with disabilities (OPDs) are established only at the regional level so there was no formal disability structure in the local context. Therefore, we worked closely with the district council social welfare office, which considers the needs of people living with disabilities, through community health workers (CHWs), and social welfare extension officers to identify households and recruit study participants.” (page 8, lines 190-195)
Lines 172-178, 210-212, 243-245: It wasn’t clear to me as I read the methods how households with a person with a disability were included in the study. At lines 172-178 it seems the 200 households are part of your analysis, but then at lines 210-212 the survey is described only for households with a member with a disability. At lines 243-245, I did not understand who the reference group for regression would be.<br><br>I think this can be clarified easily. I suggest specifying under “Sample Procedure” that the 200 households from the broader study are the reference group for your study, and under “Data Collection” clarifying what surveys were completed by the 200 control households.<br><br>Note that during my review, Figure 2 was located at the end of the document, so I did not see it as I read the Methods. Although Figure 2 makes the sampling clear, I think it would be beneficial to include that in the text as well.	**Sampling Procedures:**<br>Thank you, we have updated the Sampling Procedure section to clarify that we aimed to recruit 200 households through random sampling (as part of the wider process evaluation) with an additional 50 households with a disabled member sampled purposively.<br><br>**Data Collection:**<br>The Data Collection section has been updated to make procedures clear. The same methods were applied at all households – where no disabled member was present, surveys were only administered with one household member.	**Sampling Procedures:**<br><br>**Amended:** “Multiple sampling approaches were used in the recruitment of study participants (Fig 2). We aimed to recruit a total of 250 households; 200 households using random sampling and 50 households using purposive sampling.” (page 8, lines 178-179)<br><br>**Amended:** “Purposive sampling was used to ensure an adequate number of households with a member living with a disability were interviewed within the study timeframe in addition to the 200 households randomly sampled as part of the wider process evaluation.” (page 8, lines 187-190)<br><br>**Data Collection:**<br><br>**Amended:** “In each participating household, surveys were-first completed with the respondent identified as most responsible for

		water and sanitation in the household (Fig 3). To determine if anyone in the household had a disability, the Washington Group Short Set (WG-SS) of questions on functioning were administered with the primary household respondent [45]. Anyone in the household reported as having 'a lot of difficulty' or 'cannot do at all' in any one of the WG-SS functional domains (vision, hearing, communication, cognition, mobility, and self-care) was defined as having a disability. If a household member with a disability was present, the same survey was then completed with them. In most households where two participants were interviewed, the second respondent was the household member living with a disability. However, if the primary respondent was the household member living with a disability, the second respondent recruited from the household was an adult household member without a disability. If the second respondent was not available or not eligible as per the exclusion criteria, only the primary respondent was interviewed but the household was still noted as having a member living with a disability." (pages 9-10, lines 218-232)
Lines 247-248: There are multiple ways to account for clustering e.g, cluster-robust standard errors, random intercepts, etc. Please specify your approach briefly.	Adjustment for clustering was done using cluster robust standard errors. This has now been specified in the methods.	**Amended:** "Analyses were also adjusted for potential clustering at the village level using cluster robust standard errors." (page 11, line 256)<br><br>**Amended:** "Analyses were also adjusted for clustering of individuals within the same household using cluster robust standard

		errors. This level of clustering accounted for clustering of individuals within households" (page 11, lines 261-263).
Line 253: Presumably individual-level information is still clustered at the village level, as well as household. If you think this is correct, clustering should be accounted for at the highest level. In this case, that would be the village. Fortunately, accounting for clustering at the highest level takes care of clustering at lower levels. So only one correction is needed.<br><br>The following reference explains that this is not always the case. I expect the village-level to still be ideal in your case, but please explain if not.	Thank you, we agree the village level is likely best in our case. The individual-level analysis has been re-run and results updated to reflect this.	The updated results are presented as follows:<br><br>**Amended Abstract:**<br>"Household members without a disability felt they could give more input in triggering activities (OR=3.72, 95%CI 1.18-11.73), and reported higher participation in the transect walk (OR=4.03, 95%CI 1.45-11.18), community action planning (OR=2.89, 95%CI 1.36-6.13), and follow-up visits (OR=3.37, 95%CI 1.78-6.40) compared to household members with disabilities. There was no difference in the likelihood of being invited to triggering (OR=0.98, 95%CI 0.41–2.36), attending triggering (OR=2.09, 95%CI 0.98–4.46), or participating in community mapping (OR=2.38, 95%CI 0.71-7.98) between household members with and without a disability." (page 2, lines 42-50)<br><br>**Individual-level analysis:**<br>Participation in CLTS was proportionally lower among household members living with a disability for all activities. Individual-level regression models found no association between having a disability and being invited to triggering (OR=0.98, 95%CI 0.41–2.36), attending triggering (OR=2.09, 95%CI 0.98-4.46) or participating in community mapping (OR=2.38, 95%CI 0.71-7.98). However, household members without a disability had greater odds of participating in all other

		aspects of CLTS programming. Household members without a disability gave more input in triggering activities (OR=3.72, 95%CI 1.18-11.73), and reported higher participation in the transect walk (OR=4.03, 95%CI 1.45-11.18), community action planning (OR=2.89, 95%CI 1.36-6.13), and follow-up visits (OR=3.37, 95%CI 1.77-6.40) compared to household members living with a disability (**Error! Reference source not found.**). (page 22, lines 342-352)<br><br>**Updated Table 4:** (page 23-24, lines 355-359)
Lines 256-257: Can you explain why univariate regression was used for this subset of models, but not models of program participants? I would think that age, sex, and income would apply to all models equally.	This was done due to the smaller sample size used in these models to prevent overfitting (we only included those who attended triggering). However, following discussions with the study team, we agree it is better to keep the results consistent and the adjusted results have been presented. Pearson's goodness-of-fit test confirmed the adjusted models fit well.	**Amended:** "Multiple logistic regression was performed to calculate odds ratios to compare reporting between household members with and without a disability with adjustment for age, sex, and income. Analyses were also adjusted for clustering at the village level using cluster robust standard errors." (page 11, lines 266-270)<br><br>**Updated Table 5:** (page 26, lines 369-372)
**Results**		
Lines 283-288: I think it would be informative to also describe the demographics of households from the random sample (~200 households), which represents the broader study population, and the purposive sample (~50 households). One hundred percent of households in the purposive sample includes someone with a disability, for example, so I think it is more noteworthy to report that ~30/200 households from the random sample included an individual with a disability. The	Thank you, some wording has been added to clarify the number of households with a disabled member identified in the random sample.	**Amended:** "A total of 247 households were enrolled, of which 32% (80/247) had a member living with a disability. This included the 200 households randomly sampled and an additional 47 households purposively sampled, just short of our targeted purposive sample of 50 households. Seventeen percent

results already provided can still be included to describe the total sample, but I recommend including subgroup results as well.<br><br>Note that I believe the structure of Table 1 and Table 2 (separating any household/individual with a disability vs. without) is fine and I do not recommend separating the detailed results by sample.		(33/200) of households from the random sample had a member with a disability." (page 13, lines 298-302)
Table 3: Can you please that I have the correct interpretation of the OR for invitation to the triggering event? Households with no member with a disability were 32% less likely (aOR = 0.68, 95% CI 0.38, 1.24) to be invited to the triggering event compared to households with a member with a disability.<br><br>I want to clarify because I am surprised at the direction of the aOR, considering the proportion invited is larger for households with no member with a disability. I don't doubt it is plausible after adjusting for covariates, but it surprises me so I want to be sure.	Yes, your interpretation is correct. Without adjustment the OR = 1.04, and with adjustment the aOR changes to 0.68. The ORs are not statistically significant either way.	Table 3 (pages 20-21, lines 336-340), Table 4 (pages 23-24, lines 355-359) and Table 5 (page 26, lines 369-372) have been updated to include unadjusted odds ratios.
Overall: did you consider gender as a potential effect modifier rather than a confounder? It seems plausible to me that having a disability affects community participation, social connection, etc. differently between men and women. Also, since individuals without disabilities are so much more likely to be female (92%) than individuals with disabilities (44%), adjusting for gender may not be enough to really account for the gender distributions. I have seen situations where an adjusted estimate masks differences for heavily skewed covariates.<br><br>I would be interested in seeing how CLTS participation differs between men with disabilities and women with disabilities. The sample size may not be large enough to separately fit regression models by gender, but if sample size weren't an issue I would be interested in that as well.	Thank you for raising this. We did consider gender as a potential effect modifier, but our results showed it was not.<br><br>The gender difference in participation amongst people living with disabilities is something we are interested in exploring further, along with other factors such as disability type and severity. As you stated, we don't have sufficient sample size to look at predictors of participation amongst people with disabilities (n=55), but this is something we are interested in exploring further.	
**Discussion**		

Overall: It is unclear to me if CLTS activities were supposed to cover issues specific to individuals with disabilities, such as low-cost technologies.<br><br>Since this study was conducted in partnership with World Vision, I think it would be helpful to discuss whether disability-relevant issues were considered before implementation. The results should be interpreted differently depending on training. If CLTS facilitators failed to cover information that they were trained to include, there is a significant issue with implementation. If CLTS facilitators were not specifically instructed to cover disability-relevant information, the results demonstrate the need to specifically incorporate them into training.	Thank you, I have added some clarification in the discussion and conclusion making it clear that CLTS facilitators were not trained to deliver inclusive CLTS, so this should be incorporated in future training and guidelines.	**Added:** "Facilitators in the WASH for Everyone intervention were not specifically trained to deliver inclusive CLTS. CLTS guidelines lack specific sections addressing the needs of people living with disabilities [21]. Furthermore, national strategies and data collection tools used for ODF verification do not adequately consider disability-related factors [51, 52]". (page 28, lines 412-416).<br><br>**Added:** "While CLTS facilitators in the WASH for Everyone intervention were not specifically trained to deliver inclusive CLTS; our findings demonstrate the potential shortcomings of standard approaches and suggest that deliberate action is needed to ensure true community-wide participation. The omission of disability-inclusive activities from CLTS guidelines impacts the participation of people living with disabilities in CLTS initiatives. Addressing this gap is essential for inclusive sanitation practices and ensuring no community member is left behind." (page 30-31, lines 467-473)

**Author edits**
Minor changes to wording and grammar (not mentioned above) have been made and are highlighted in tracked changes.
