# Supplementary material for: Inclusion of persons living with disabilities in a district-wide sanitation programme: A cross-sectional study in rural Malawi
Source: PLOS Glob Public Health. 2024 Aug 29;4(8):e0003005. doi: 10.1371/journal.pgph.0003005 (PMC11361574; doi:10.1371/journal.pgph.0003005)
Supplement: Attachment — Submitted filename: Response to Reviewers_31.07.24.pdf [file pgph.0003005.s007.pdf]

31 July 2024

Dear Editor,

Thank you for inviting us to submit a revised draft of our manuscript, "**Inclusion of persons living with disabilities in a district-wide sanitation programme: a cross-sectional study in rural Malawi",** to PLoS Global Public Health. We thank you for providing insightful feedback on how to strengthen our paper. We have amended the manuscript to address these comments, with detailed responses to each comment provided on the following page.

Thank you for considering our manuscript for publication. We look forward to your response.

Sincerely,

Katherine Davies

Reviewer comment	Response	Action/revision
**Editor Comments**		
• Describe the methods used to assess effect modification by gender (subgroup analysis or use of interaction terms, if the latter, the p-value cut-off for defining significant effect modification) as well as the numerical results from this analysis.<br>• In the results section, please report the gender breakdown among individuals with vs. without disabilities in the text and include ORs and p-values from the effect modification analysis in the text and/or in a table.<br>• Please also comment on how the small stratum of males without disabilities may have affected the effect modification analysis.	• Thank you, I have clarified in the methods that a model with an interaction term for gender was fitted to assess effect modification. I have also added a sentence clarifying a p-value cut-off of <0.05 was used.<br>• The gender breakdown has been added to the beginning of the results section. A table outlining the p-values from the effect-modification analysis has been added to the supporting information (Table S1).<br>• Commentary has been added to the limitations about how the small stratum of males without disabilities may have affected the analysis.	• Models with interaction terms for gender were fitted to test if gender was an effect modifier in the relationship between disability and CLTS participation. The significance of effect modification was determined using a p-value cut-off of <0.05 (page 11, lines 265-268)<br>• Respondents who did not have a disability were more likely to be female (92%; 208/226) than male (8%; 18/226). The gender distribution was more even among respondents with a disability who were more likely to be male (56%; 31/55) than female (44%; 24/55). (page 13, lines 318-321).<br>• Our analysis exploring effect modification did not find evidence of interaction between gender and disability. However, we note the limited number of male respondents who did not have a disability. These results should be interpreted with caution. Future studies that are adequately powered to explore the combined effects of gender and disability are needed. (page 30-31, lines 455-460).
Please replace singular conjugations following data (e.g., "data was") with plural conjugations (e.g., "data were") throughout the text.	This has been updated throughout the manuscript.	
